# Retinal guanylyl cyclase activating protein 1 forms a functional dimer

**DOI:** 10.1371/journal.pone.0193947

**Published:** 2018-03-07

**Authors:** Sunghyuk Lim, Graham Roseman, Igor Peshenko, Grace Manchala, Diana Cudia, Alexander M. Dizhoor, Glenn Millhauser, James B. Ames

**Affiliations:** 1 Department of Chemistry, University of California, Davis, CA, United States of America; 2 Department of Chemistry and Biochemistry, University of California, Santa Cruz, CA, United States of America; 3 Pennsylvania College of Optometry, Salus University, Elkins Park, PA, United States of America; Russian Academy of Medical Sciences, RUSSIAN FEDERATION

## Abstract

Retinal guanylyl cyclases (RetGCs) in vertebrate photoreceptors are regulated by the guanylyl cyclase activator proteins (GCAP1 and GCAP2). Here, we report EPR double electron-electron resonance (DEER) studies on the most ubiquitous GCAP isoform, GCAP1 and site-directed mutagenesis analysis to determine an atomic resolution structural model of a GCAP1 dimer. Nitroxide spin-label probes were introduced at individual GCAP1 residues: T29C, E57C, E133C, and E154C. The intermolecular distance of each spin-label probe (measured by DEER) defined restraints for calculating the GCAP1 dimeric structure by molecular docking. The DEER-derived structural model of the GCAP1 dimer was similar within the experimental error for both the Mg^2+^-bound activator and Ca^2+^-bound inhibitor states (RMSD < 2.0 Å). The GCAP1 dimer possesses intermolecular hydrophobic contacts involving the side chain atoms of H19, Y22, F73 and V77. The structural model of the dimer was validated by GCAP1 mutations (H19R, Y22D, F73E, and V77E) at the dimer interface that each abolished protein dimerization. Previous studies have shown that each of these mutants either diminished or completely suppressed the ability of GCAP1 to activate the cyclase. These results suggest that GCAP1 dimerization may affect compartmentalization of GCAP1 in the photoreceptors and/or affect regulation of the cyclase activity.

## Introduction

Retinal guanylyl cyclase activator proteins (GCAP1[1] and GCAP2[2]) are the two most ubiquitous among the animal species EF-hand calcium sensor proteins in vertebrate photoreceptor rod and cone cells[3]. Both GCAP1 and GCAP2 control Ca^2+^-sensitive activation of retinal guanylyl cyclases (RetGCs[4–7]) that is crucial for promoting the recovery phase of the photoresponse [6, 8]. Visual excitation causes hydrolysis of cGMP in photoreceptor cells and promotes closure of cGMP-gated channels[9, 10], which lowers the cytosolic Ca^2+^ concentration from ~250–500 nM in the dark down to <50 nM in the light[11, 12]. The light-induced decrease in Ca^2+^ causes the formation of Ca^2+^-free/Mg^2+^-bound GCAPs to activate RetGC[13], whereas Ca^2+^-bound GCAPs inhibit RetGC at high Ca^2+^ levels maintained in the dark[14–16]. Mutations that impair Ca^2+^ binding to GCAP1 cause constitutive activation of RetGC[17–25] and these GCAP mutations are genetically linked to retinal degenerative diseases, dominant cone- and cone-rod degenerations [26]. Animal models expressing constitutively active GCAP1 mutants linked to the diseases demonstrate that the congenital blindness is triggered by abnormally high cGMP and intracellular Ca^2+^ concentrations resulting from the deregulation of RetGC by GCAPs[11, 27].

**Fig 1:**
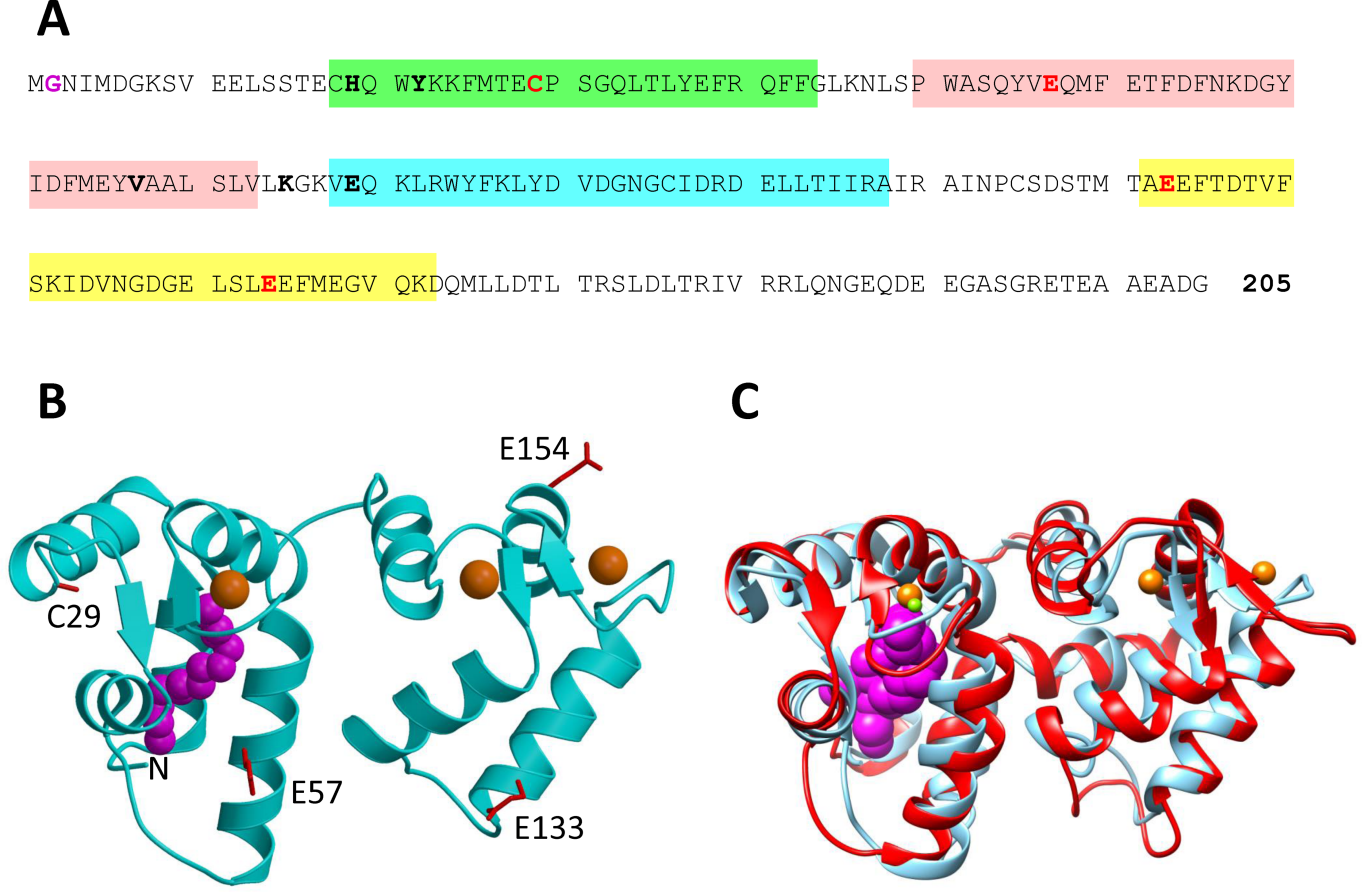
Primary and tertiary structure of GCAP1. (A) Amino acid sequence of bovine GCAP1, (B) crystal structure of GCAP1 in the Ca^2+^-saturated state (cyan, PDB code: 2R2I), and (C) overlay of GCAP1 crystal structure (cyan) with NMR Structure of GCAP1 in the Ca^2+^-free/Mg^2+^-bound state (red, PDB code: 2NA0). EF-hand motifs in the primary sequence are shaded in color (EF1 green, EF2 red, EF3 cyan and EF4 yellow). GCAP1 residues substituted with cysteine (T29C, E57C, K67C, E133C, E154C) that are attached to a nitroxide spin-label in DEER studies are highlighted in bold and red. Key residues at the dimer interface are highlighted bold and black. N-terminal myristoyl group is colored magenta. Bound Ca^2+^ and Mg^2+^ are colored orange and green, respectively.

The structure of GCAP1 contains 4 EF-hands (EF1, EF2, EF3 and EF4) and N-terminal myristoylation (Fig 1A). The atomic-level structures of GCAP1 indicate relatively small Ca^2+^-induced changes in tertiary structure (Fig 1B and 1C). The crystal structure of Ca^2+^-saturated GCAP1 (cyan in Fig 1B and 1C) and the NMR structure of Ca^2+^-free/Mg^2+^-bound GCAP1 (magenta in Fig 1C) have an overall RMSD < 1.8 Å. In addition to Ca^2+^ binding, GCAPs undergo dimerization [28–30] that may play a role in regulating RetGC, which also forms a dimer [31, 32]. Ca^2+^-sensitive protein dimerization was reported for recoverin[33], neurocalcin[34] and GCAP2[30, 35] that are structurally quite similar to GCAP1. We hypothesize that quaternary structural changes in a GCAP1 dimer may allosterically regulate RetGC activity and thus amplify the small Ca^2+^-induced changes in tertiary structure of GCAP1 akin to the allosteric regulation of O_2_ binding to hemoglobin[36].

In the current study, we performed EPR DEER analysis to calculate an atomic level structural model of the GCAP1 dimer. Nitroxide spin-label probes were introduced at four different sites in GCAP1 (T29C, E57C, E133C and E154C, see Fig 1B) and intermolecular distances from each site in the GCAP1 dimer were measured by DEER. The DEER distance restraints define a GCAP1 dimer structure that contains key hydrophobic residues (H19, Y22, F73, V77 and W94) at the dimer interface. Site-directed mutagenesis analysis reveals that key amino acids mutated at the dimer interface (H19R, Y22D, F73E, V77E, and W94A) each weaken protein dimerization and drastically reduce cyclase activation. We propose that GCAP1 dimerization may facilitate its binding to dimeric RetGC in a 2:2 complex, and cyclase activation by GCAP1 may involve allosteric conformational changes in quaternary structure analogous to the R to T transition in hemoglobin[36].

## Materials and methods

### Expression and purification of GCAP1

Recombinant bovine myristoylated GCAP1 was used throughout this study and bacterial expression of myristoylated GCAP1 was accomplished by co-expressing the GCAP1 D6S mutant and yeast N-myristoyl CoA transferase (NMT) in E. coli strain, BL21(DE3) as described previously[29]. Purification of GCAP1 was achieved using previously described methods [28, 37]. Typically, 10 mg of final purified GCAP1 protein was obtained from 1 liter of cell culture. The final protein sample was more than 95% pure as judged by SDS-PAGE.

### GCAP1 mutagenesis

Mutations in cDNA coding for a D6S bovine GCAP1 were introduced using a ‘splicing by overlap extension technique’[38] as previously described in detail[39]. To produce Cys-less mutant of GCAP1 (**GCAP1**^**CL**^), all original Cys residues in GCAP1 were substituted by Thr. Subsequent introduction of single and double Cys residues was done using the GCAP1^CL^ cDNA as a template. The resultant cDNA constructs were ligated into the NcoI/BamHI sites of pET11d vector (Novogen/Calbiochem) for subsequent expression in E. coli as described above.

### Analytical size exclusion chromatography (SEC)

The molar mass of GCAP1 and mutants in the presence of saturating Mg^2+^ (2 mM) or Ca^2+^ (2 mM) was determined using analytical SEC (Superdex 200 HR 10/30 column, GE Healthcare). The calibration procedure was described by [40]. A sample volume of 100 μL (200 μM protein concentration) was applied to the column equilibrated with standard sample buffer (10 mM Tris (pH 7.4), 100 mM NaCl, and 1 mM dithiothreitol) at 4°C with a flow rate of 0.5 ml/min.

### NMR spectroscopy

Samples of GCAP1 and mutants for NMR experiments consisted of ^15^N-labeled myristoylated and Mg^2+^-bound GCAP1 (0.5 mM) concentrated in 10 mM Tris (pH 7.4) buffer containing 2 mM MgCl_2_, 1 mM DTT-d_10_ and 90%:10% H_2_O:D_2_O. All NMR experiments were performed at 37°C on a Bruker 800 MHz Avance III spectrometer equipped with a triple resonance cryogenic TCI probe and pulsed field gradients. 2D ^15^N-^1^H HSQC spectra were recorded with 2048 (^1^H) x 256 (^15^N) data points.

### Spin labeling and DEER sample preparation

Protein samples were dialyzed against 4 L dialysis buffer in 20 mM Tris (pH 7.4) with 100 μM TCEP overnight at 4°C and diluted to 20 μM. The spin-label (1 oxyl 2,2,5,5-tetramethyl-Δ3-pyrroline-3-methyl) methanethiosulfonate spin-label (MTSSL, Toronto Research Chemicals Inc., Toronto, Canada) was dissolved in dimethylsulfoxide (DMSO) to a concentration of 40 mM. Excess MTSSL was added to protein at a 50:1 molar ratio and then reacted on ice for 30 min. Unreacted spin-labels were removed by dialyzing overnight at 4°C against 4 L dialysis buffer containing 20 mM Tris (pH 7.4) and repeated twice. The protein was concentrated to final concentration of ~300 μM by ultrafiltration using Amicon spin concentrator. The final protein sample for DEER experiments was exchanged three times with 10 mM Tris-d_11_ (pH 7.4), 2 mM MgCl_2_ and 99.9% D_2_O. Before sample freezing, 25% glycerol-d_8_ was added to the protein as a cryo-protectant.

### EPR-DEER measurements

4-pulse DEER data was collected on a Bruker ELEXSYS E580 X-band spectrometer equipped with an MD-5 dielectric resonator and a second frequency DEER module (Bruker). The pump pulse was fixed to the center peak in the field swept nitroxide spectrum and the probe frequency was chosen 65–75 MHz away from this frequency. π/2 and π pulses were 16 and 32 ns, respectively. The delay between the first and second probe pulses was 400 ns and dipolar evolution data was collected out to 2.5–4.0 μs. Experiments were run at 50 or 80 K, depending on the sample, and were signal averaged for 8–24 hr. The raw data was background corrected and analyzed by Tikhonov regularization using the LongDistances program processed with LabView^TM^. CW-EPR spectra of the DEER samples were recorded at 123 K with a Bruker EMX EPR spectrometer operating at the X-band frequency (~9.4 GHz) using an ER 4122SHQE resonator (Bruker).

### Retinal guanylyl cyclase assays

The human RetGC1 cDNA was expressed in HEK293 cells from a modified pRCCMV vector (Invitrogen) using calcium phosphate precipitation for the transfection, and the membrane fraction containing expressed RetGC1 was isolated and assayed for activity as previously described in detail[13]. Briefly, the assay mixture (25 μL) incubated at 30°C contained 30 mM MOPS–KOH (pH 7.2), 60 mM KCl, 4 mM NaCl, 1mM DTT, 2 mM Ca^2+^/EGTA buffer, 1 mM free Mg^2+^, 0.3 mM ATP, 4 mM cGMP, 1 mM GTP, and 1 μCi of [α-^32^P]GTP. The resultant [^32^P]cGMP product was separated by TLC using fluorescently-backed polyethyleneimine cellulose plates (Merck) developed in 0.2 M LiCl, eluted with 2 M LiCl and the radioactivity was counted using ScintiSafe liquid scintillation cocktail containing 20% ethanol.

### Molecular docking calculation

The web-based docking program HADDOCK[41] was used to generate a structural model of the GCAP1 dimer using the NMR structure of bovine GCAP1 V77E mutant (PDB ID: 2NA0) as a template structure. Intermolecular distance restraints derived from DEER measurements on GCAP1 mutants (GCAP1^CL^(E57C), GCAP1^CL^(E133C), and GCAP1^CL^(E154C)) served as input for the docking calculation. Since the DEER intermolecular distance distribution for E57C showed two maxima at 26 and 50 Å, two separate calculations were performed using the following intermolecular distance restraints. For the first calculation (symmetric dimer in Table 1), the following intermolecular distance restraints were used: 50 Å (E57C), 50 Å (E133C), and 28 Å (E154C). A second calculation (asymmetric dimer in Table 1) used 26 Å (E57C), 50 Å (E133C), and 28 Å (E154C). An error margin of ±5 Å was applied to each restraint. Table 1 lists the distance restraints and HADDOCK parameters used in each calculation.

**Table 1:** Molecular docking statistics for GCAP1.

	Symmetric Dimer	Asymmetric Dimer
DEER distance restraint	E57C Sγ: 50 ± 5 Å	E57C Sγ: 26 ± 5 Å
DEER distance restraint	E133C Sγ: 50 ± Å	E133C Sγ: 50 ± Å
DEER distance restraint	E154C Sγ: 28 ± Å	E154C Sγ: 28 ± Å
Calculated distance	E57C Sγ: 46 Å	E57C Sγ: 36 Å
Calculated distance	E133C Sγ: 55 Å	E133C Sγ: 45 Å
Calculated distance	E154C Sγ: 35 Å	E154C Sγ: 29 Å
HADDOCK Energy	-181.3 ± 5.8	-133.8 ± 5.5
RMSD (Å)^a^	0.6 ± 0.3	0.7 ± 0.4
Cluster size	30	47

^a^root mean squared deviation of backbone heavy atoms.

The docking calculation within HADDOCK used rigid body energy minimization that generated 5000 structures. The best 200 structures were subjected to a semi-flexible simulated annealing step. In the final step, the 200 structures obtained from the simulated annealing step were refined in explicit waters. The 200 water refined structures were classified into 12 clusters (symmetric dimer) and 3 clusters (asymmetric dimer) as shown in S1 Fig. The cluster having the lowest interface RMSD (i-RMSD) was chosen for analysis and the statistics of this cluster are summarized in Table 1.

## Results

### Intermolecular distances of a GCAP1 dimer by EPR-DEER

**Fig 2:**
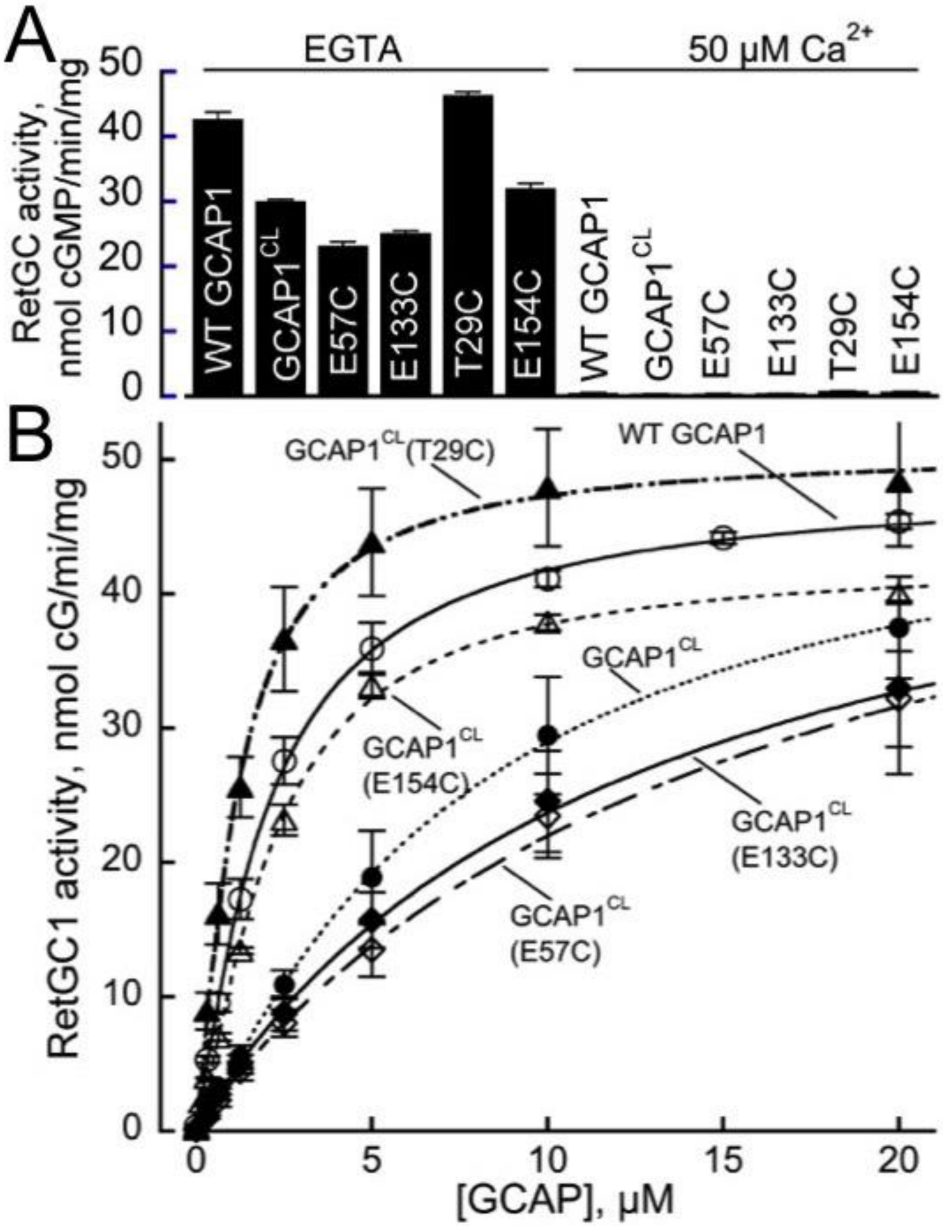
Single-Cys GCAP1 mutants retain the ability to regulate RetGC. Membranes from HEK 293 cells expressing guanylyl cyclase RetGC1 were reconstituted with the purified recombinant myristoylated GCAP1 and assayed as described under Materials and Methods. (A) RetGC1 activity after reconstitution with 10 μM wild type, Cys-less GCAP1^CL^ and single-Cys mutants measured in the presence of either EGTA (<10 nM free Ca^2+^) or 50 μM free Ca^2+^; mean average ± SD from 3 measurements. (B) Dose-dependence of the cyclase activation in the presence of EGTA by wild type GCAP1 (○), a Cys-less mutant GCAP1^CL^(●) or mutants containing a single Cys, GCAP1^CL^(T29C) (▲), GCAP1^CL^(E57C) (◇), GCAP1^CL^(E133C(◆), and GCAP1^CL^(E154C) (△); mean average ± SD from 3 independent experiments.

Previous studies demonstrated that GCAP1 forms a dimer in solution[28, 29]. In this study, we analyzed EPR-DEER measurements to calculate a structural model of the GCAP1 dimer. DEER experiments were performed on GCAP1 and were analyzed to measure intermolecular distances between individual nitroxide spin-labels covalently attached to particular Cys residues on the protein surface (T29C, E57C, E133C, and E154C). Wild type GCAP1 contains four native Cys residues (C18, C29, C106 and C125) that in principle could be spin-labeled. Unfortunately, the attachment of a spin-label at each native Cys interfered with GCAP1 dimerization or caused protein aggregation. Therefore, in subsequent mapping of the intermolecular intractions by DEER, we eliminated the native Cys residues by replacing them with Thr and then introduced a single Cys in various positions. Each native Cys residue was mutated to Thr to produce a Cys-less mutant, GCAP1^CL^. Since Cys^29^, unlike all other Cys residues in GCAP1, is a part of the RetGC-binding interface[39], the original C29T substitution in the GCAP1^CL^ reduced affinity of the Cys-less protein for the cyclase compared to the wild type (EC_50_ of 14 vs < 2 μM, respectively), but the GCAP1^CL^ was still able to strongly activate the cyclase at higher concentrations in the absence of Ca^2+^ (Fig 2). Single Cys mutants of GCAP1 were generated by introducing a Cys residue in place of a particular exposed residue in the Cys-less GCAP1 (GCAP1^CL^(T29C), GCAP1^CL^(E57C), GCAP1^CL^(E133C), and GCAP1^CL^(E154C)). Each of the single Cys mutants were then individually labeled with a nitroxide spin label, and the intermolecular distance between the attached spin label was measured for each mutant using DEER. The GCAP1 Cys-less mutant and each of the single-Cys mutants (GCAP1^CL^(T29C), GCAP1^CL^(E57C), GCAP1^CL^(E133C), and GCAP1^CL^(E154C)) were all shown to be functional and capable of activating RetGC in a Ca^2+^-sensitive manner (Fig 2); however, RetGC activation by these mutants required higher GCAP1 concentrations (EC_50_ = 5–14 μM) compared to wild type (EC_50_ < 2 μM), except for the GCAP1^CL^(T29C), which activated RetGC1 in a manner indistinguishable from the wild type, because the original Cys^29^ in the cyclase-binding interface became restored in that mutant.

**Fig 3:**
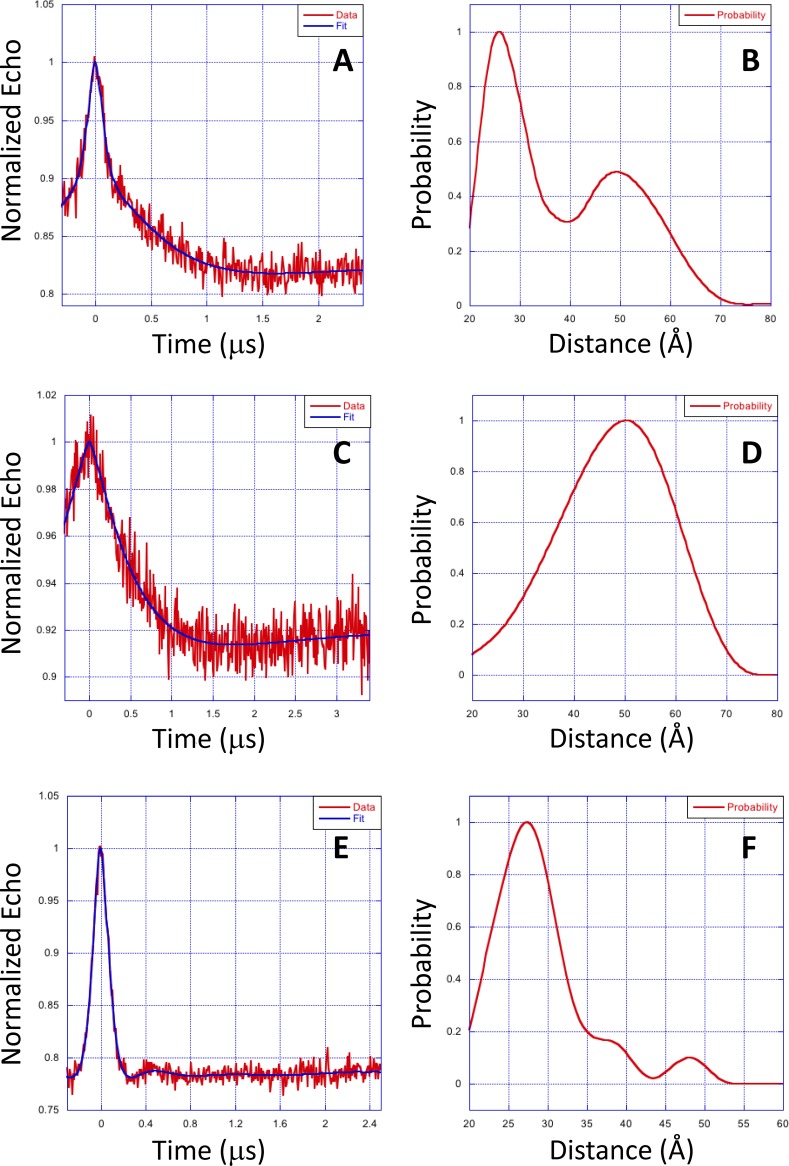
EPR-DEER intermolecular distances for GCAP1. Representative EPR-DEER data of GCAP1^CL^(E57C) (A), GCAP1^CL^(E133C) (C), and GCAP1^CL^(E154C) (E), and corresponding distance distributions of GCAP1^CL^(E57C) (B), GCAP1^CL^(E133C) (D), and GCAP1^CL^(E154C) (F). GCAP1 samples were in the Ca^2+^-free/Mg^2+^-bound state. Similar DEER data were observed for GCAP1 in the Ca^2+^-bound state. A nitroxide spin-label (MTSSL) was covalently attached to the sole Cys residue in each mutant. The distance distributions and average intermolecular distances were calculated on the basis of the DEER data as described in Methods. The bimodal distance distribution for GCAP1^CL^(E57C) suggest there are two populations of GCAP1 dimers with distinct structure near E57C. The DEER intermolecular distances were measured to be 26±3/50±5 Å (GCAP1^CL^(E57C) in panel B), 50 ±5 Å (GCAP1^CL^(E133C) in panel D), and 28 ±3 Å (GCAP1^CL^(E154C) in panel F).

Representative DEER data for each nitroxide-labeled GCAP1 single Cys mutant (GCAP1^CL^(E57C), GCAP1^CL^(E133C) and GCAP1^CL^(E154C)) are shown in Fig 3. The DEER data for GCAP1^CL^(E57C) (Fig 3A) was best modeled by a bimodal distance distribution (Fig 3B) that has two most probable intermolecular distances at 26 ±5 Å and 50 ±5 Å. The bimodal distance distribution suggests there may be two populations of structurally distinct GCAP1 dimers. The DEER data for GCAP1^CL^(E133C) (Fig 3C) was accurately modeled by a single distance distribution (Fig 3D) with a most probable intermolecular distance of 50 ±5 Å. The DEER data for GCAP1^CL^(E154C) (Fig 3E) was fit to a single distance distribution (Fig 3F) with a most probable intermolecular distance of 28 ±3 Å. The measured DEER intermolecular distances are listed in Table 1.

**Fig 4:**
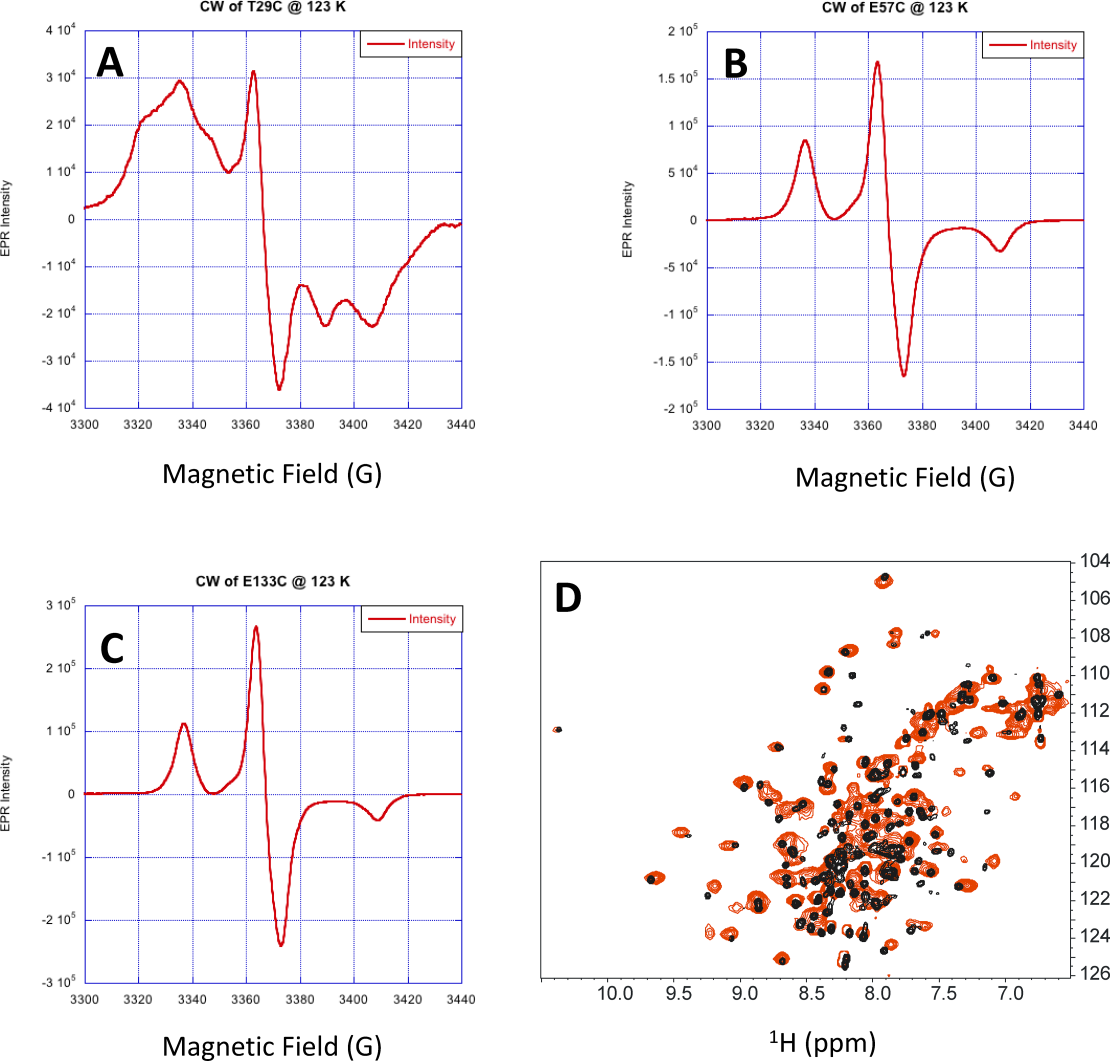
Spin-label attached to GCAP1^CL^(T29C) has restricted motion. CW-EPR spectra of GCAP1^CL^(T29C) (A), GCAP1^CL^(E57C) (B) and GCAP1^CL^(E133C) (C) at 123 K. (D) NMR ^1^H-^15^N HSQC spectra of ^15^N-labeled GCAP1^CL^(T29C) containing an attached spin-label with the N-O group reduced (red) and wildtype GCAP1 (black). Spectral parameters are described in Methods.

The intermolecular distance for GCAP1^CL^(T29C) could not be accurately measured by DEER, because of apparent strong dipolar coupling of the nitroxide spin label attached at T29C. Evidence of strong dipolar coupling of the nitroxide at this site is evident in the CW-EPR spectrum of GCAP1^CL^(T29C) (Fig 4A) that exhibited a broad shoulder and multiplet lineshape. By contrast, the CW-EPR spectra of GCAP1^CL^(E57C) (Fig 4B) and GCAP1^CL^(E133C) (Fig 4C) exhibited well-resolved and sharp resonances, consistent with a solvent exposed nitroxide with an intermolecular distance of greater than 20 Å [42]. The strong dipolar coupling of the nitroxide at T29C would imply a spin-label intermolecular distance of less than 9 Å. However, such a short intermolecular distance for T29C is inconsistent with the intermolecular DEER distances measured above for GCAP1^CL^(E57C), GCAP1^CL^(E133C), and GCAP1^CL^(E154C) (Fig 3). We hypothesized that the 9 Å intermolecular distance for T29C might be the result of higher order protein multimers (eg a dimer of dimers) that is not seen in the wild type protein. Indeed, the HSQC NMR spectrum of GCAP1^CL^(T29C) containing an attached spin-label (with the N-O group reduced [43]) has considerably broader NMR peaks compared to wild type GCAP1 (Fig 4D). Also, NMR pulsed-field gradient diffusion studies [44] on spin-labeled GCAP1^CL^(T29C) determined a translational diffusion coefficient (D = 2.0 x 10^−10^ m^2^/s) consistent with a molar mass of ~100 kDa, which corresponds to a protein tetramer for the spin-labeled GCAP1^CL^(T29C) in contrast to the dimeric wild type protein [29]. These results suggest that the strong dipolar coupling observed for the spin-labeled GCAP1^CL^(T29C) may be the result of protein tetramerization and/or aggregation that is not seen in the wild type protein dimer. Therefore, the strong dipolar coupling of the nitroxide attached at T29C is possibly an artifact of protein aggregation and will not be included in the dimeric structural model below.

### Structural model of a GCAP1 dimer

**Fig 5:**
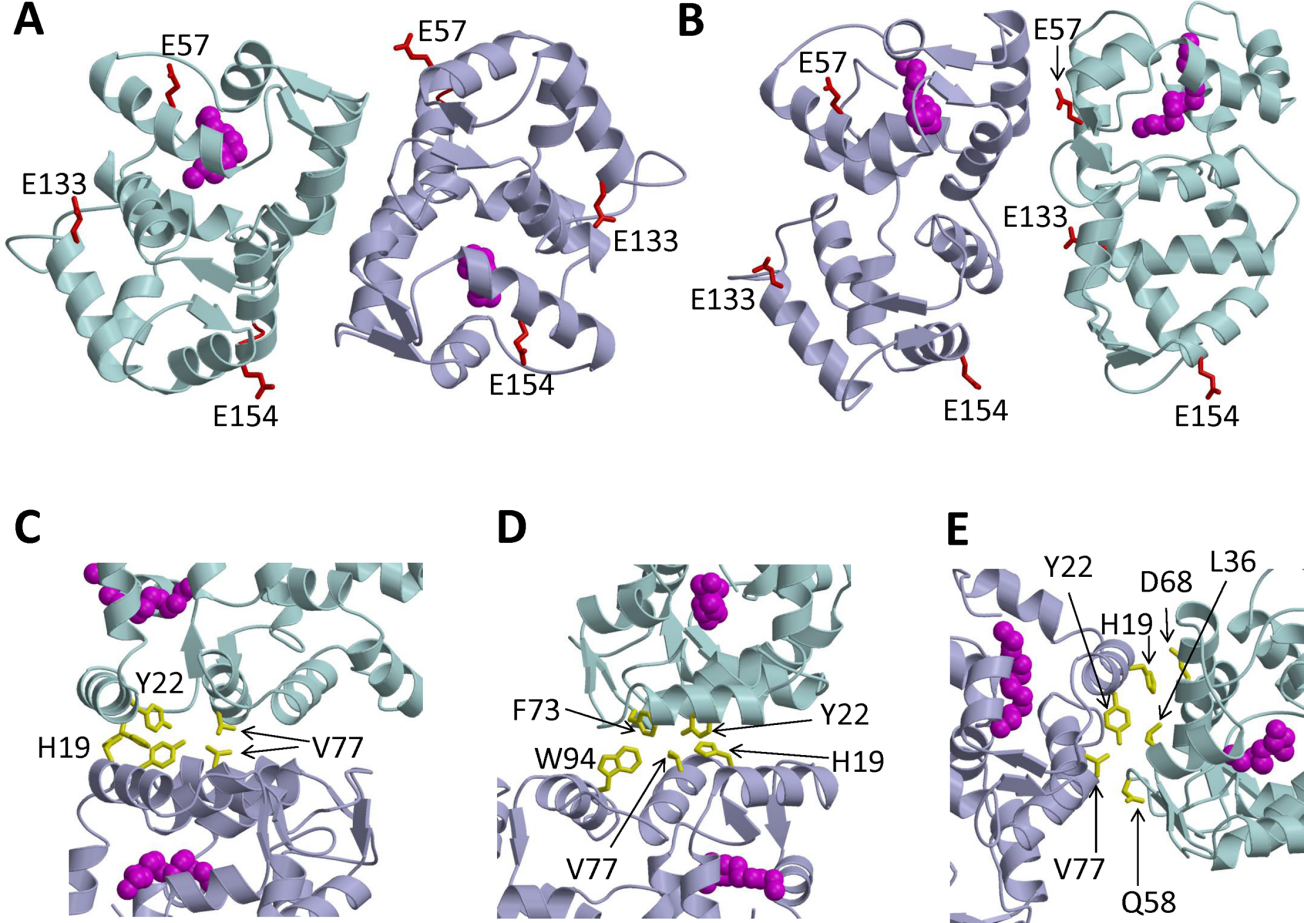
Structural model of GCAP1 dimer. Ribbon diagram of main chain structures of the GCAP1 symmetric dimer (A) and asymmetric dimer (B) that are both consistent with DEER intermolecular distances for spin-label attached to E57C, E133C and E154C. Exposed residues mutated to Cys for DEER studies (E57, E133 and E154) are colored red in panels A and B. Myristoyl group is highlighted magenta. (C) Close-up view of the symmetric dimer showing hydrophobic residues (side-chains of H19, Y22, and V77 colored yellow) at the dimer interface. (D) Close-up view of the symmetric dimer showing intermolecular hydrophobic contacts between Y22, F73, V77 and W94. (E) Close-up view of the asymmetric dimer showing hydrophobic residues (H19, Y22, and V77 colored yellow) at the dimer interface.

The intermolecular distances for each of the spin-labeled GCAP1 mutants measured by DEER (Fig 3) were used as distance restraints within HADDOCK[45] to calculate the structure of the GCAP1 dimer as described in Methods. The bimodal DEER distance distribution for GCAP1^CL^(E57C) (Fig 3B) suggests that the two distance components may represent two different GCAP1 dimer structures. Therefore, the distance restraints (intermolecular distances between spin labels attached at E57C, E133C and E154C in Table 1) were analyzed to calculate two separate structural models that represent each distance component for GCAP1^CL^(E57C). One of the models is a symmetric dimer that has a E57C intermolecular distance matching the long distance component at ~50 Å (Fig 5A). The other model is an asymmetric dimer that has the E57C intermolecular distance matching the shorter distance component at 26 Å (Fig 5B).

The GCAP1 symmetric dimer (Fig 5A) is stabilized structurally by intermolecular hydrophobic contacts at the dimer interface. The most central contacts are formed between H19, Y22 and V77 (Fig 5C). The V77 side-chain methyl groups make intermolecular contact with each other at the dimer interface (Fig 5C). The intermolecular contact involving V77 is particularly important, which explains why the V77E mutation abolished GCAP1 dimerization[29]. The aromatic side-chains of H19 and Y22 also make intermolecular contacts with each other at the dimer interface (Fig 5C). The symmetric dimer is further stabilized by intermolecular hydrophobic contacts between aromatic side chains of F73 and W94 (Fig 5D). The symmetric GCAP1 dimer (Fig 5A) is structurally quite similar to the dimeric structural model reported recently for GCAP5 [40].

The GCAP1 asymmetric dimer (Fig 5B) has both hydrophobic and polar residues at the dimer interface. The hydrophobic residues observed at the dimer interface in the symmetric dimer (H19, Y22, V77 and W94) are also located at the dimer interface in the asymmetric dimer (Fig 5E). Surprisingly, the hydrophobic residues (H19 and V77) both make intermolecular contacts with polar residues at the dimer interface. The side chain methyl groups of V77 form intermolecular contacts with the negatively charged side chain of D68, and the aromatic imidazole side chain of H19 forms intermolecular contacts with the side chain atoms of Q58 (Fig 5E). The asymmetric dimer is also stabilized by intermolecular hydrophobic contacts between the aromatic ring of Y22 and methyl groups of L36 (Fig 5E).

**Fig 6:**
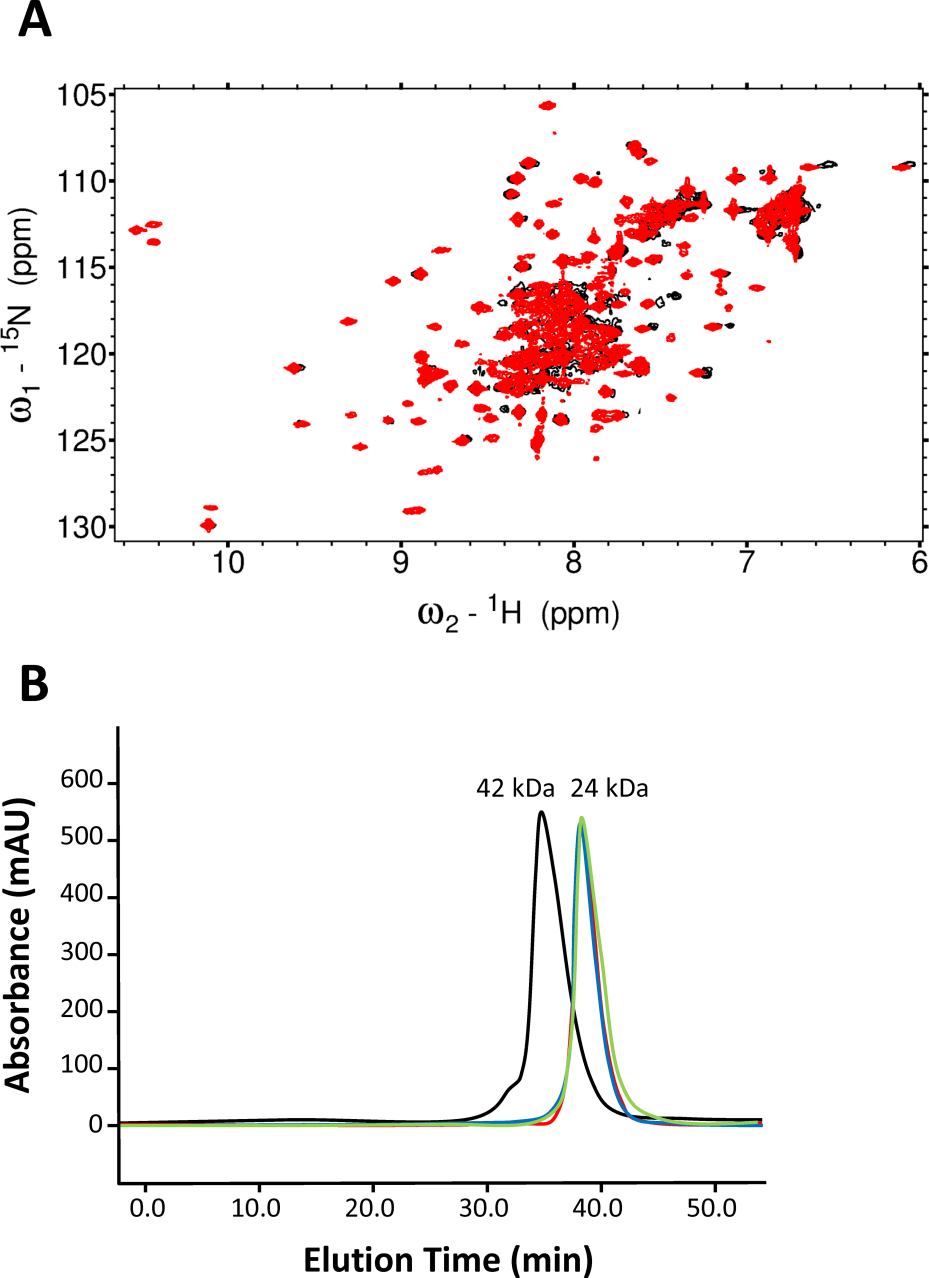
Mutations at the GCAP1 dimer interface abolish dimerization. (A) ^1^H-^15^N HSQC NMR spectra of ^15^N-labeled wild type GCAP1 (black) and GCAP1 mutant, F73E (red) in the Ca^2+^-bound state. The NMR peaks of the F73E mutant are sharper and more sensitively detected than the corresponding peaks from wild type GCAP1. The conditions for the NMR experiments were the same as described previously[29]. (B) SEC analysis of GCAP1 mutants: H19R (red), Y22A (blue), F73E (green), and wild type (black). The elution time of wildtype GCAP1 in the Mg^2+^-bound state corresponded to a molar mass of 42 ±4 kDa (relative to protein standards), and the molar mass of each mutant was calculated to be 24 ±2 kDa. The addition of saturating Ca^2+^ or Mg^2+^ had no effect on the elution times. The SEC experimental conditions are described in Methods.

To discriminate the two dimeric GCAP1 structures (symmetric vs asymmetric), we constructed a mutant (F73E) that is predicted to disrupt key intermolecular hydrophobic contacts between F73, V77 and W94 in the symmetric dimer (Fig 5D), whereas the F73E mutation is predicted to have less of an effect on the asymmetric dimer. The ^1^H-^15^N HSQC NMR spectrum of ^15^N-labeled F73E mutant (red peaks in Fig 6A) exhibited a greater number of much sharper NMR peaks compared to those of wildtype GCAP1 (black peaks in Fig 6A), which were broader and less sensitively detected. The sharper and narrower NMR peaks observed for the F73E mutant suggest that the mutant protein is a monomer in contrast to the dimeric wild type protein. In addition, the F73E mutation was shown previously to dramatically decrease its ability to activate RetGC[46]. The effect of the F73E mutation on both protein dimerization and cyclase activation suggests that the symmetric dimer (Fig 5A) is biologically relevant.

### Mutations at GCAP1 dimer interface abolish dimerization

To validate the structures of the GCAP1 dimer (Fig 5A and 5B), residues at the dimer interface were each mutated (H19R, Y22D, F73E and V77E). The molecular size of each GCAP1 mutant in solution was determined by size exclusion chromatography (Fig 6B). The elution time of wild type GCAP1 corresponded to a molar mass of 42 kDa, indicating that wild type GCAP1 is a dimer in solution at protein concentrations above 100 μM[29]. The addition of saturating Ca^2+^ and/or Mg^2+^ had no detectable effect on the elution time of wild type GCAP1 or any of the mutants. The elution times of each mutant (H19R, Y22D, F73E and V77E) corresponded to a molar mass of 24 kDa, demonstrating that each mutant is a monomer in solution. Therefore, mutating each of the key residues at the dimer interface (H19R, Y22D, F73E and V77E) significantly weakened the dimerization association constant, which validates the structural models of the GCAP1 dimer (Fig 6A and 6B).

## Discussion

We present EPR-DEER (Figs 3 and 4) and mutagenesis functional analysis (Figs 2 and 6) to demonstrate that GCAP1 forms a dimer (Fig 5), which we suggest can be functionally important for cyclase activation. The structure of the GCAP1 dimer is summarized in Fig 5. A cluster of hydrophobic residues (H19, Y22, F73 and V77) form important intermolecular contacts at the dimer interface that greatly stabilize the dimer (Fig 5C and 5D). Site-directed mutagenesis of the residues at the dimer interface (H19R, Y22D, F73E, and V77E) each abolished dimerization (Fig 6) and prevented activation of RetGC[46]. We reason that dimeric GCAP1 may interact with the RetGC dimer to form a 2:2 complex, and quaternary structural changes in the dimer may play a role in regulating the cyclase activity during visual phototransduction.

Previous mutagenesis studies have mapped a cluster of residues on the surface of GCAP1 that are critical for cyclase activation [39, 47, 48]. Many of these GCAP1 residues that are essential for activating the cyclase (H19, Y22, F73, and V77) are also located at the GCAP1 dimer interface (Fig 5C and 5D). One possible interpretation is that dimerization of GCAP1 (Fig 5) may be a prerequisite for it to bind and activate the cyclase. Indeed, the mutations in this study that each abolished dimerization (H19R, Y22D, F73E and V77E) also prevented activation of RetGC [39]. This suggests that a pre-formed GCAP1 dimer may facilitate its binding to the dimeric cyclase and GCAP1 dimerization could help stabilize a high affinity 2:2 target complex.

**Fig 7:**
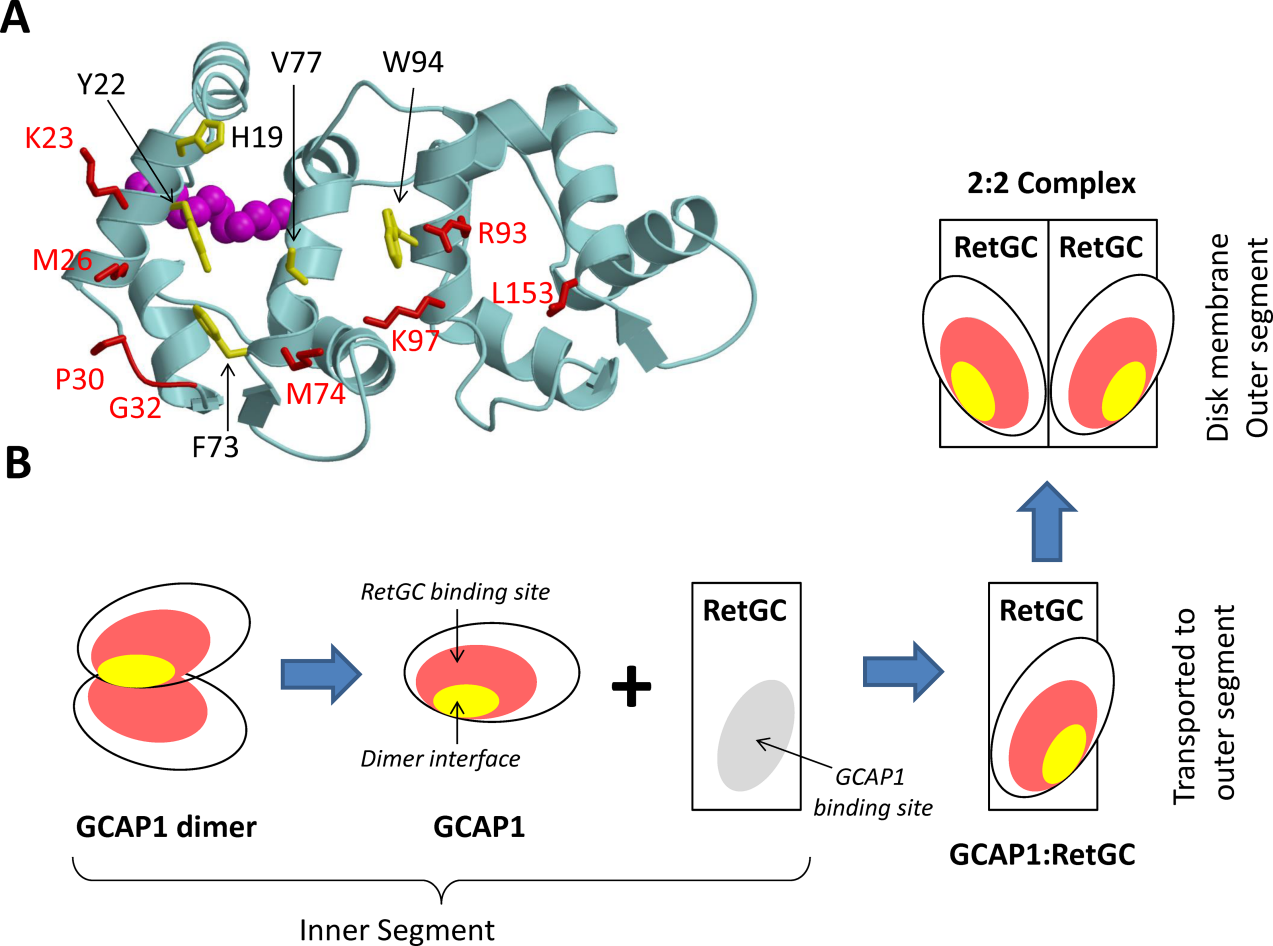
A hypothetical function of GCAP1 dimerization in photoreceptor cells. (A) The RetGC-binding site (red) and dimerization interface (yellow) in GCAP1 overlap. The residues essential for regulation of the cyclase[39] are highlighted in red and those that are required for dimerization of GCAP1 in solution are highlighted in yellow. (B) GCAP1 (in excess of RetGC) in the inner segment forms a dimer via its dimerization interface, which prevents GCAP1 diffusion into the outer segment. The binding of RetGC to GCAP1 disrupts the GCAP1 dimer, which allows the RetGC/GCAP1 complex to incorporate in transport vesicles that are then transported to the outer segment. In the outer segment, the RetGC/GCAP1 complex is translocated into disk membranes, where it forms a 2:2 complex.

An alternative possibility is that the GCAP1 dimer that forms in solution (and in the absence of RetGC) may dissociate upon binding to membrane-bound RetGC (Fig 7). Mutations that disrupt GCAP1 dimerization also either drastically (H19R) or completely (Y22D, F73E, and V77E) suppress the ability of GCAP1 to activate its primary target enzyme, RetGC1[39]. The last three mutations also prevent the binding of GCAP1 to RetGC1, which was probed by fluorescently labeled RetGC1 and GCAP1 co-expressed in the same cells[39]. Hence, the dimer interface partially overlaps with the cyclase binding site[39], with several residues (His^19^, Tyr^22^, Phe^73^, Val^77^ and Trp^94^) being part of both interfaces (Fig 7A). In this scenario, the residues at the GCAP1 dimerization site may prefer to interact more strongly with RetGC (rather than itself) once GCAP1 is in close proximity to the target enzyme. The binding of RetGC to GCAP1 in this context would be expected to disrupt GCAP1 dimerization in favor of binding with the cyclase (Fig 7B). The exact concentration of GCAP1 in the inner segment, and especially its local concentration at the points of assembly with the cyclase is impossible to estimate at present, but we hypothesize that dimerization of GCAP1 in the absence of the target enzyme could explain the inability of GCAP1 to efficiently accumulate in the rod outer segments lacking RetGC1[49]. According to this hypothesis, only GCAP1 associated with the RetGC would be carried into the outer segment by a vesicle transport mechanism that delivers the membrane-bound cyclase to the photoreceptor disks. By contrast, the passive diffusion of cytosolic dimeric GCAP1 (that is not bound to the cyclase) into the outer segment may be restricted by the size of the GCAP1 dimer (Fig 7B). To distinguish whether GCAP1 dimerization facilitates or opposes RetGC binding, future EPR-DEER studies are needed to probe whether or not the structure of the GCAP1 dimer (Fig 5) will remain intact when GCAP1 is bound to RetGC. Additional *in vivo* studies of GCAP1 and RetGC1 co-transport are also needed to elucidate compartmentalization of RetGC *vs*. dimeric or the monomeric of GCAP1 in the photoreceptor cell.

## Supporting information

S1 Fig Cluster analysis of HADDOCK docking calculation. (PDF)
