# Supplementary material for: Retinal guanylyl cyclase activating protein 1 forms a functional dimer
Source: PLoS One. 2018 Mar 7;13(3):e0193947. doi: 10.1371/journal.pone.0193947 (PMC5841803; doi:10.1371/journal.pone.0193947)

S1 Figure. Cluster Analysis of HADDOCK Docking Calculation. HADDOCK z-score plotted against i-RMSD for (A) symmetric dimer and (B) asymmetric dimer. The cluster with the lowest i-RMSD was chosen for analysis and described in Table 1.

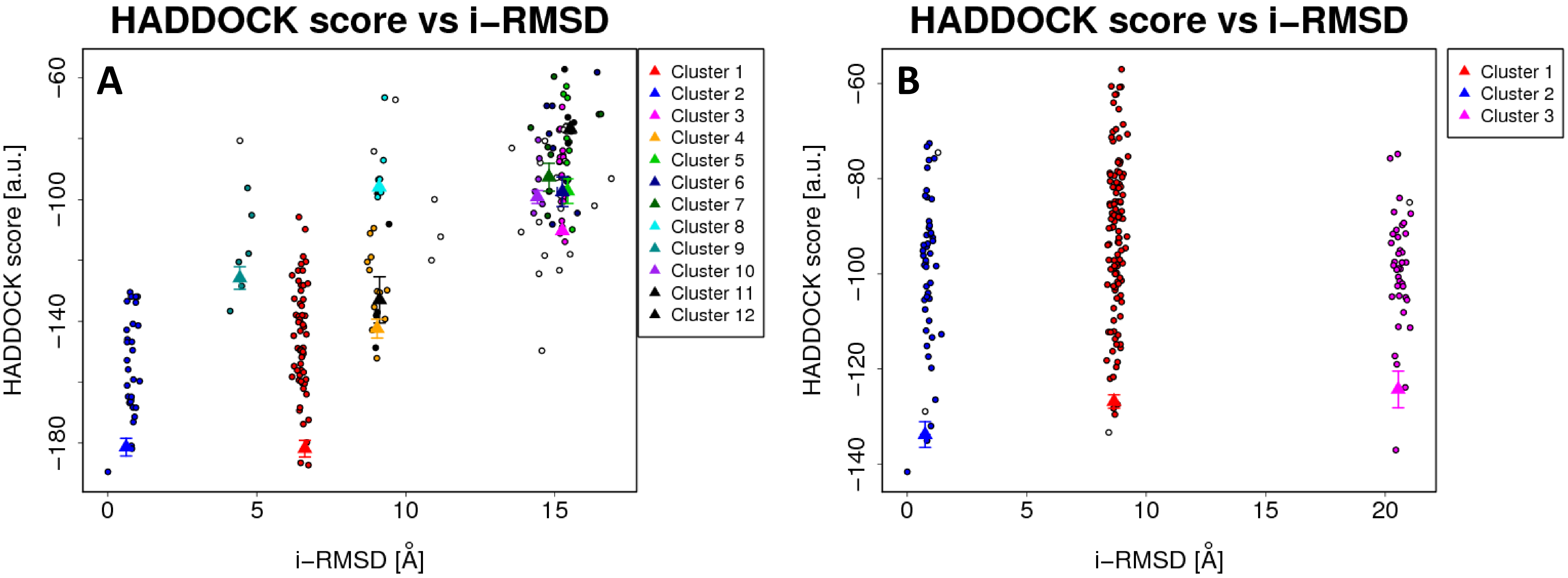

Supplement: S1 Fig — (PDF) [file pone.0193947.s001.pdf]
